# Rethinking of Alzheimer's disease: Lysosomal overloading and dietary therapy

**DOI:** 10.3389/fnagi.2023.1130658

**Published:** 2023-02-13

**Authors:** Shu Yuan, Si-Cong Jiang, Zhong-Wei Zhang, Yu-Fan Fu, Xin-Yue Yang, Zi-Lin Li, Jing Hu

**Affiliations:** ^1^College of Resources, Sichuan Agricultural University, Chengdu, China; ^2^Haisco Pharmaceutical Group Comp. Ltd., Chengdu, China; ^3^Department of Cardiovascular Surgery, Xijing Hospital, Medical University of the Air Force, Xi'an, China; ^4^School of Medicine, Northwest University, Xi'an, China

**Keywords:** Alzheimer's disease, ketogenic diets, lysosomal overloading, trehalose, unsaturated fatty acids introduction

## Introduction

The amyloid precursor protein (APP) is infamous for its putatively critical role in the pathogenesis of Alzheimer's disease (AD) (Kim et al., 2022; Lee et al., 2022; Öhman et al., 2022; Thorwald et al., 2022). However a recent study found that autolysosome acidification declines in neurons age more than 4 months before amyloid β-protein (Aβ) deposited extracellularly (Lee et al., 2022). Endolysosome de-acidification increases intraneuronal and secreted levels of Aβ (Hui et al., 2019). On the other hand, autolysosome acidification increases the degradation of accumulated Aβ in autophagic vacuoles (AVs; Nie et al., 2018) and promotes glial clearance of oligomeric amyloid-β (oAβ; Huang et al., 2022). Therefore, autolysosome acidification declines directly result in Aβ aggregation. APP accumulates selectively within enlarged and de-acidified lysosomes. In more compromised yet still intact neurons, profuse Aβ-positive AVs pack into large membrane tubules. Then lysosomal membrane permeabilization, cathepsin release and lysosomal-mediated cell death occur, accompanied by microglial invasion (Lee et al., 2022). Thus, Aβ accumulation may be the “result” rather than the “cause”. The finding prompts rethinking of the conventionally accepted sequence of AD plaque formation and may help explain the inefficiency of Aβ/amyloid vaccines and Aβ/amyloid-targeted therapies (Lee and Nixon, 2022).

## Lysosomal overloading hypothesis

**Figure 1:**
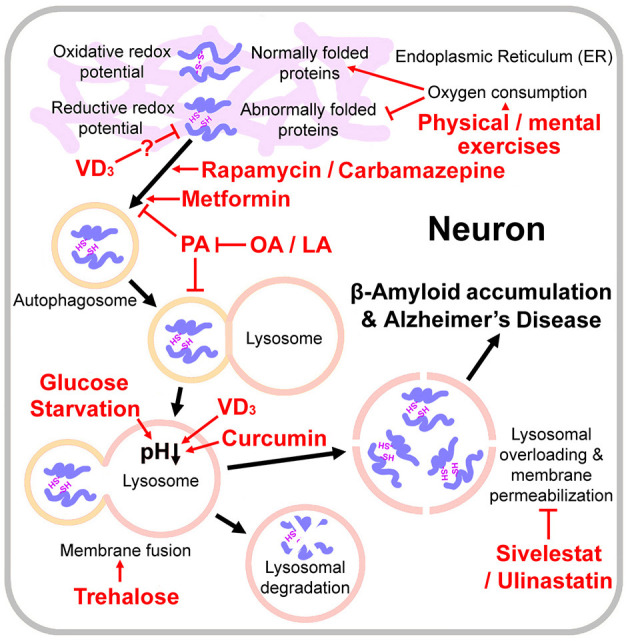
Pathogenesis of Alzheimer s disease and the relevant drugs and dietary therapies. Physical / mental exercises generate the oxidative redox potential required to oxidize the free sulfydryl groups of cysteine into the disulphide bonds for proper protein folding. Lysosomal overloading (misfolded protein over-accumulation) induces lysosomal membrane permeabilization, amyloid β-protein accumulation and Alzheimer's disease onset. VD_3_ activates protein disulfide isomerase family A member 3 and thus may reduce misfolded proteins. Rapamycin, carbamazepine and metformin are autophagy stimulators. Glucose starvation, trehalose, VD_3_, and curcumin promote autophagosome-lysosome fusion and/or lysosomal acidification. Lysosome integrity protectants Sivelestat and Ulinastatin may also protect neurons from lysosomal-mediated cell death. Palmitic acid (PA) causes autophagy impairment; however, oleic acid (OA) or linoleic acid (LA) counteracts PA's detrimental effects on neurons.

It is well-known that patients with type II diabetes have increased probability of developing AD (Watson, 2014; Hamzé et al., 2022; van Arendonk et al., 2022). In 2014, James D Watson proposed that type II diabetes is a redox disease (Watson, 2014). The diabetic cells may have higher reductive redox potentials than non-diabetic cells. Insulin resistance and type II diabetes may develop through insufficient supplies of key reactive oxygen species (ROS) that creates an oxidative redox potential required to oxidize the free sulfydryl groups of cysteine into the disulphide bonds used to stabilize the 3D conformation of key proteins controlling blood sugar levels. Physical exercise, by generating ROS, reverses the reductive redox potential in diabetic cells and therefore alleviates the progression of diabetes (Watson, 2014). We speculate that there may be a similar pathogenic mechanism for AD. Like type II diabetes, as Alzheimer's disease progresses, the endoplasmic reticulum (ER) of cells in stressed hippocampal regions largely accumulate unfolded or misfolded proteins (Hoozemans et al., 2009). Although the formation of inter-molecular disulfide bond may accelerate aggregation of Aβ fibrils, introducing intra-molecular disulfide bonds at certain positions inhibited Aβ fibrillization (Shivaprasad and Wetzel, 2004). Pathogenesis of AD depends on the balance between the rate of misfolded protein production and the rate of lysosomal clearance. This assumption has been proved by a mouse experiment: activated neural stem cells (NSCs) had active proteasomes; while quiescent NSCs contained large but inactive lysosomes. During aging, quiescent NSCs showed defects in their lysosomes and increased accumulation of peptide aggregates (Leeman et al., 2018). While physical and mental exercises enhance oxygen consumption and may generate the oxidative redox potential needed for proper folding of these proteins (Figure 1). A prospective cohort study indicated that frequent exercises, housework-related activities, and friend/family visits were associated with a reduced risk of multiple types of dementia (Guo et al., 2022; Zhu et al., 2022).

Enhancement of the autophagic/lysosomal pathway may be an effective solution to promote clearance of misfolded proteins and prevent the occurrence of AD (Figure 1). Rapamycin, a well-known autophagy stimulator, has beneficial effects in a number of animal models of neuro-degeneration including AD (Kaeberlein and Galvan, 2019). However it is also an immunosuppressant and may increase a risk of infection for the old people. Carbamazepine an mTOR (mechanistic target of rapamycin kinase)-independent autophagy stimulator, prevented the compromised autophagic flux in septic mouse liver (Lin et al., 2014) and restored the mitophagic flux caused by a decline in lysosomal acidification (Ebrahimi-Fakhari et al., 2016; Chan et al., 2022). However, it is an antiepileptic drug and may not be applicable to all AD patients. Metformin treatments activated chaperone-mediated autophagy and improved disease pathologies in an AD mouse model (Xu et al., 2021). However, the available clinical evidences do not support the idea that metformin could reduce risk of AD (Luo et al., 2022), which may be because of complex effects of metformin on cellular metabolic process pathways.

Lysosome lysis occurs and the proteases are released, causing β-Amyloid accumulation and neuron death (Lee et al., 2022). Thus the lysosome integrity protectants (elastase inhibitors) should be also considered for the AD treatment, such as Ulinastatin (Yamasaki et al., 1996) and Sivelestat (Iwata et al., 2010) (Figure 1). However, no clinical data about therapeutic effect of either of them are available so far. Such clinical trials should be undertaken.

In general, both autophagy-stimulators and lysosomal acidifiers showed therapeutic effects to neurodegenerative diseases by increasing clearance of pathologic proteins, such as Aβ in AD and α-synuclein (α-syn) in Parkinson's disease (PD; Moors et al., 2017; Limanaqi et al., 2021; Perez et al., 2022). However, broad stimulation of autophagy may cause a wide spectrum of dose-dependent side-effects, suggesting that its clinical applicability is limited (Moors et al., 2017). The targeted manipulation of downstream autophagy-lysosomal-pathway components (Moors et al., 2017), or some natural active components (see below for details), may be attractive strategies for the development of novel pharmacological therapies in neurodegenerative diseases.

## Dietary therapy hypothesis

Early features of AD includes a region-specific decrease in brain glucose metabolism, which may affect brain function profoundly (Henderson, 2008; Taylor et al., 2019; Lilamand et al., 2021). One promising treatment is to supplement the normal sugar supply of the brain with ketone bodies (KB). KB are usually produced from fat stores when glucose supply is limited, e.g. prolonged fasting. KB can be generated through the administration of low-carbohydrate, low-protein, high-fat, ketogenic diets (Henderson, 2008). Although high-fat diets may not be suitable for the people with hyperlipidemia, human trials reported significantly cognitive benefits, improved brain metabolism and biomarker changes under ketogenic diets (Taylor et al., 2019; Lilamand et al., 2021).

On the other hand, beneficial effects of ketogenic diets on AD may also be attributed to the enhanced lysosomal activity induced by glucose starvation. Glucose starvation promotes the assembly of a lysosomal AMPK (AMP-activated protein kinase) activating complex, consisting of V-ATPase, AMPK, liver kinase B1 (LKB1), AXIN (Axis inhibitor), aldolase, and Ragulator-RAG (Ras-related GTPase), and then induces lysosomal acidification mediated by V-ATPase (González et al., 2020).

Besides glucose, trehalose also regulates autophagy and lysosomal functions. Trehalose, a glucose disaccharide with a flexible α-1-1'-glycosidic bond, induces mTOR-independent autophagy by promoting AMPK pathways and functions as a chaperone on proteins folding (Pupyshev et al., 2022) (Figure 1). In a mouse model of amyotrophic lateral sclerosis (ALS), trehalose rescued impaired autophagosome-lysosome fusion caused by the disease, and therefore significantly delayed onset of the disease (Zhang et al., 2014; Yuan et al., 2017). And in an AD mouse model, trehalose reduced colocalization of APP and β-site amyloid precursor protein cleaving enzyme 1 (BACE1) in the neuron (Benito-Cuesta et al., 2021). Nevertheless, a large part of trehalose would be broken down into glucose by trehalase on the intestinal mucosa (Richards et al., 2002). It is still unclear how much trehalose should be taken by oral administration to reach an effective serum concentration. We suggest that a part of carbohydrates in ketogenic diets could be replaced by trehalose to achieve a synergistic inducing effect on lysosomal acidification.

Vitamins may also be supplied in the ketogenic diet. Vitamin D_3_ (VD_3_) promoted lysosomal degradation through inducing the nuclear translocation of PDIA3 (protein disulfide isomerase family A member 3) - STAT3 (signal transducer and activator of transcription 3) protein-complex and up-regulated the MCOLN3 (mucolipin 3) channel subsequently, which resulted in Ca^2+^ releasing from the lysosome and the normal lysosomal acidification (Hu et al., 2019; Chan et al., 2022). VD_3_ and VE and their combination improved memory and learning deficit, and decreased neuronal loss and oxidative stress in a rat model of AD (Mehrabadi and Sadr, 2020). It is interesting to note that PDIA3 also acts as a chaperone, responsive to ER stress, to facilitate correct disulfide bond formation and protein folding (Mahmood et al., 2021). Thus, VD_3_ may be helpful to reduce misfolded proteins in neurons and relieve lysosomal burden from the source (Figure 1), although this assumption needs further experimental validation.

Disorder of lipid metabolism, especially when the palmitic acid (PA) accumulates, would cause lipid-overload lipotoxicity and inhibit the autophagic flux in neurons, which is the whole autophagic process from the synthesis of the autophagosomes to their lysosomal fusion and degradation (Fang et al., 2020; Hernández-Cáceres et al., 2020; Chung, 2021; Vesga-Jiménez et al., 2022). PA induces APP palmitoylation and enhances Aβ accumulation via proteolytic processing of APP by multiple enzymes, such as BACE1, β-secretase, and γ-secretase (Kim et al., 2017; Zareba-Kozioł et al., 2018). A recent study demonstrated that, the Silence information regulator 3 (SIRT3), an NAD^+^ dependent deacetylase enzyme regulating multiple mitochondrial proteins, is also involved in the neuro-inflammation during the onset of AD (Tyagi et al., 2021). A combination of high glucose and PA treatments resulted in a significant decline in expression level of SIRT3. And in *Sirt3*-silenced mouse brain-derived endothelial cells, the neuro-inflammatory response was exacerbated (Tyagi et al., 2021). Many researchers proposed the use of unsaturated fatty acids (UFA), such as oleic acid (OA) or linoleic acid (LA), as a potential therapeutic approach against AD, because that these UFA could counteract PA's detrimental effects on cells (Piomelli, 2013; Vesga-Jiménez et al., 2022). For example, oleic acid ingestion stimulates oleoylethanolamide mobilization into the mucosal cells of the gut, which triggers a peroxisome proliferator-activated receptor (PPAR)-mediated signals that travel through the afferent vagus nerve to the hypothalamus, augmenting satiety and showing beneficial effects to the nervous system (Piomelli, 2013).

Some plant natural products are also autophagy stimulators, such as berberine and curcumin (Figure 1). The combination of berberine and curcumin treatment reduced the APP and BACE1 levels and increased AMPK phosphorylation and autophagy (Lin et al., 2020). However, berberine is a broad-spectrum antibiotic, and a long-term use may easily lead to an intestinal flora disorder. Curcumin repressed mTORC1 signaling in by two mechanisms involving deactivation of IRS-1 (insulin receptor substrate-1) and excitation of AMPK (Kaur and Moreau, 2021). Curcumin also promoted autophagosome-lysosome fusion (Kang et al., 2019) and inhibited tau protein hyper-phosphorylated oligomerization, which is another pathogenic mechanism of AD (Rane et al., 2017). Curcumin is relatively safe, but its stability and pharmacokinetics are low (at neutral or alkaline pH, the oral bioavailability of curcumin in rats is less than 1%; Nelson et al., 2017). Nevertheless, a study with healthy human volunteers found that concomitant administration of 20 mg piperine increased bioavailability of curcumin (at a dose of 2 g) by 20 times (Shoba et al., 1998). Hereby, turmeric powder and pepper powder may also be added in the ketogenic diet.

## Conclusions and perspectives

In summary, during aging, misfolded proteins accumulate, especially when physical and mental exercises are lacking. When the accumulation level reaches a level beyond the clearance capacity of lysosomes, Aβ accumulation and AD occur. Some autophagy-stimulating drugs or lysosomal acidifiers showed therapeutic effects to AD. Besides, ketogenic diets with low-carbohydrate administration may have neuro-protective benefits to AD patients. Trehalose, VD_3_, unsaturated fatty acids (UFA), curcumin may also be incorporated into the ketogenic diet. If merely dietary regulation with over-the-counter drugs (like VD_3_) can achieve significantly curative effects on AD, it would be a particularly attractive research direction. However, the relevant studies are just beginning, and a large number of clinical trials are warranted. Nevertheless, for the patients with middle and late stage AD, dietary therapies may not be very effective, and therefore highly potent and relatively safe autophagy stimulators or lysosome activators still need to be developed.

## Author contributions

SY conceived the project. S-CJ, Z-WZ, Y-FF, and X-YY performed the literature search. SY wrote the manuscript with input from S-CJ, Z-WZ, Y-FF, X-YY, Z-LL, and JH. All authors contributed to the article and approved the submitted version.
